# Zirconium-89-labelled rituximab PET-CT in orbital inflammatory disease

**DOI:** 10.1186/s13550-019-0530-9

**Published:** 2019-07-30

**Authors:** Kamil G. Laban, Rachel Kalmann, Roos J. Leguit, Bart de Keizer

**Affiliations:** 1 Department of Ophthalmology, University Medical Center Utrecht, Utrecht University, Room E 03.136, P.O. Box 85500, 3508 GA Utrecht, The Netherlands; 2 Laboratory of Translational Immunology, University Medical Center Utrecht, Utrecht University, Utrecht, The Netherlands; 3 Department of Pathology, University Medical Center Utrecht, Utrecht University, Utrecht, The Netherlands; 4 Department of Radiology and Nuclear Medicine, University Medical Center Utrecht, Utrecht University, Utrecht, The Netherlands

**Keywords:** Idiopathic orbital inflammation, Thyroid eye disease, 89Zr-rituximab PET/CT, Rituximab

## Abstract

**Background:**

Orbital inflammatory diseases are a heterogenic group of conditions that often entail a difficult diagnostic process and many patients are treatment resistant. Inflammatory diseases can be visualized by Zirconium-89-labelled rituximab PET-CT (^89^Zr-rituximab PET/CT). In this study, we describe our experience and possible potential of the ^89^Zr-rituximab PET/CT for diagnostic and therapeutic management of refractory orbital inflammation.

**Results:**

Retrospectively, ^89^Zr-rituximab uptake was assessed and related to clinical data. The main outcome measures were the characteristics of the scan and the clinical relation of uptake with the diagnostic process and treatment effectivity. Twelve patients with thyroid eye disease (TED) and suspected idiopathic orbital inflammation (IOI) were scanned. Six patients had a strong ^89^Zr-rituximab uptake and showed a focal distribution within the lesion. Four patients (one TED, three IOI) responded well to rituximab treatment after a positive scan. ^89^Zr-rituximab PET/CT was essential to the diagnosis of optic nerve meningioma in one patient.

**Conclusion:**

^89^Zr-rituximab PET/CT has the potential to be a powerful tool for the detection of B cell-mediated disease within the orbit and ocular adnexa. This technique can be a valuable addition for diagnosing diseases around the eye and can potentially predict rituximab treatment response in patients with refractory inflammation.

## Background

Orbital inflammatory disease comprises of several inflammatory conditions around the eye with different underlying causes [1]. The most common and well-studied cause is thyroid-associated eye disease (TED), while non-thyroid associated orbital inflammation can be a diagnostic challenge and most are considered idiopathic orbital inflammation (IOI) [2, 3]. The most important challenge is the differentiation from malignant entities, especially lymphoid malignancies, because of the grave therapeutic consequences. Currently, the best diagnostic tool available is to perform a biopsy of the orbital process with immunohistochemistry [3]. A biopsy is, unfortunately, not always deemed possible because of deep localization behind the eye and the related risk of complications [4]. From a pathological point of view, biopsies of orbital inflammation often reveal a lymphoplasmacytic infiltrate consisting of (cluster of differentiation (CD) 20+) B cells, CD5+ T cells and polytypical plasma cells. Imaging modalities that can directly detect elements of the pathophysiology of the disease, such as CD20+ B cells, may therefore have potential as an aid in the diagnostic process and management strategies. CD20+ B cell infiltrates have previously been visualized in patients with immune diseases and lymphoma with Zirconium-89-labelled (^89^Zr) rituximab positron emission tomography-computed tomography (PET-CT) [5–7]. The use of ^89^Zr-rituximab PET/CT in orbital disease has not yet been investigated. Here, we describe our experience and the potential of this technique in aiding in the diagnosis of refractive orbital inflammation.

## Methods

In this retrospective study, we included 12 patients with an ^89^Zr-rituximab PET/CT in the University Medical Center Utrecht for ophthalmologic pathology. The scans were performed because of suspected orbital inflammatory disease refractory to standard treatment. In five patients, the use of rituximab was considered as alternative treatment and it was given in four patients. At the time of the scan, all patients were rituximab naïve. The standard therapy for IOI and TED consisted of oral prednisone regimen (60 mg) tapered over 3 months or intravenous (IV) methylprednisolone (500–1000 mg/day for 3 days depending on the severity of disease), with the continuation of oral prednisone or IV methylprednisolone regimen in case of insufficient response. Refractory to standard therapy was defined as intolerance, failure to respond to, or inability to taper oral prednisone treatment, the use of multiple IV methylprednisolone regimens or systemic immunosuppressive treatment (adapted from Suhler et al. [8]). IOI was diagnosed by assessing clinical indicators, MRI imaging and an extensive laboratory panel and whenever possible a biopsy [9]. A biopsy for diagnostic confirmation was possible for all IOI except for one IOI located within the orbital apex (case 1). One patient was diagnosed with IgG4-related orbital disease (IgG4+ ROD). The research team investigated clinical data, laboratory workup, and histopathology.

### Patients

From the 12 patients (detailed description in Table 1), 8 were diagnosed with IOI, 2 patients with TED, 1 patient with IgG4+ ROD, and 1 patient ultimately with an optic nerve meningioma. The patients were refractory to standard treatment in the following way: two patients (cases 5 and 6) received oral prednisolone treatment for more than 12 months with inability for tapering. The other 10 patients did not respond to treatment with oral prednisolone and 1 or multiple intravenous (IV) methylprednisolone treatments (range 1–6 IV methylprednisolone regimens). Four patients (cases 2, 3, 11 and 12) were given additional immunosuppressive treatment (either azathioprine 100–150 mg, tocilizumab 800 mg or methotrexate 20 mg/week). Both patients with TED did not respond to multiple IV methylprednisolone regimens and retained a clinical activity score (CAS) with a score of four (case 4) and five (case 10). Due to the nature of refractory orbital inflammation, all patients were either under oral prednisolone treatment at the time of the scan or were recently given oral or IV steroids.

**Table 1:** Representation of the cases for diagnosis and PET/CT intensity values

Case	Final diagnosis	Location	Biopsy	Pain	RAPD	Proptosis (Hertel in mm)	BCVA	PET SUVmax lesion	PET SUVmax bone marrow	PET SUV max LN level 2
1	IOI	Apex with posterior extension	–	Moderate	No	Yes (24–20)	0.6	1.04 (moderate)	2.07	11.36
2	IOI	Myositis	+	Severe	No	Yes^a^	1.0	0.68 (low)	2.01	7.97
3	IOI	Lacrimal gland	+	Moderate	No	No	1.2	3.88 (high)	5.32	11.32
4	TED	Pan-myositis	–	None	No	Yes (23–23)	1.0	0.33 (low)	2.91	5.45
5	IgG4+	Myositis	+	Mild	No	Yes (16–24)	1.2	1.58 (moderate)	4.43	8.90
6	IOI	Diffuse mass	+	Moderate	No	Yes^a^	0.9	3.11 (high)	3.73	15.87
7	IOI	Diffuse mass	+	Moderate	No	Yes^a^	1.0	2.12 (high)	3.27	14.45
8	Meningioma	Apex	–	None	Yes	No	0.6	0.79 (low)	2.49	5.81
9	IOI	Diffuse mass	+	Severe	Yes	No	0.5	3.82 (high)	3.64	12.12
10	TED	Pan-myositis	–	Mild	No	Yes (29–29)	0.7	3.47 (high)	4.07	12.10
11	IOI	Myositis	+	Severe	No	No	0.9	4.24 (high)	3.52	13.71
12	IOI	Myositis	+	Severe	No	No	1.0	0.68 (low)	4.83	19.15

*IOI* = idiopathic orbital inflammation; *TED* = thyroid eye disease; *RAPD* = relative afferent pupillary defect; *BCVA* = best-corrected visual acuity

^a^Clinical and radiological proptosis, not quantified with Hertel

### ^89^Zr-rituximab PET/CT procedure

Seventy-four megabecquerel ^89^Zirconium (with a half-life of 78.4 h) was produced and labelled to 10 mg rituximab according to the procedures described previously [10]. No adverse effect occurred on administration of ^89^Zr-rituximab. Three days after intravenous administration, we performed a PET/CT of the head on a TruePoint Biograph mCT40 scanner (Siemens, Erlangen, Germany). We performed a low dose CT scan using Care Dose 4D and Care kV, reference parameters: 40 mAs, 120 kV. Subsequently, the PET was acquired according to the European Association of Nuclear Medicine (EANM) recommendations with a single bed position of 10 min with the following parameters: PET with time-of-flight and point spread function (TrueX) reconstruction, 4 iterations, 21 subsets, with a filter of 7.5 mm full width at half maximum [11]. We used tonsillar, submandibular, submental, pre- and post-auricular, and occipital lymph nodes as a positive control of CD20+ (B cell) targeting. For standardized uptake value (SUV) measurements, we used the lean body mass-corrected formula. We regarded a quantification of the PET-positivity (maximal SUV) above 2.0 to be a strong positivity, consistent with the PET-positivity of lymph nodes and bone marrow in the head and neck area, and a maximum SUV between 1.0 and 2.0 as moderate uptake.

## Results

### PET-CT analysis

**Fig. 1:**
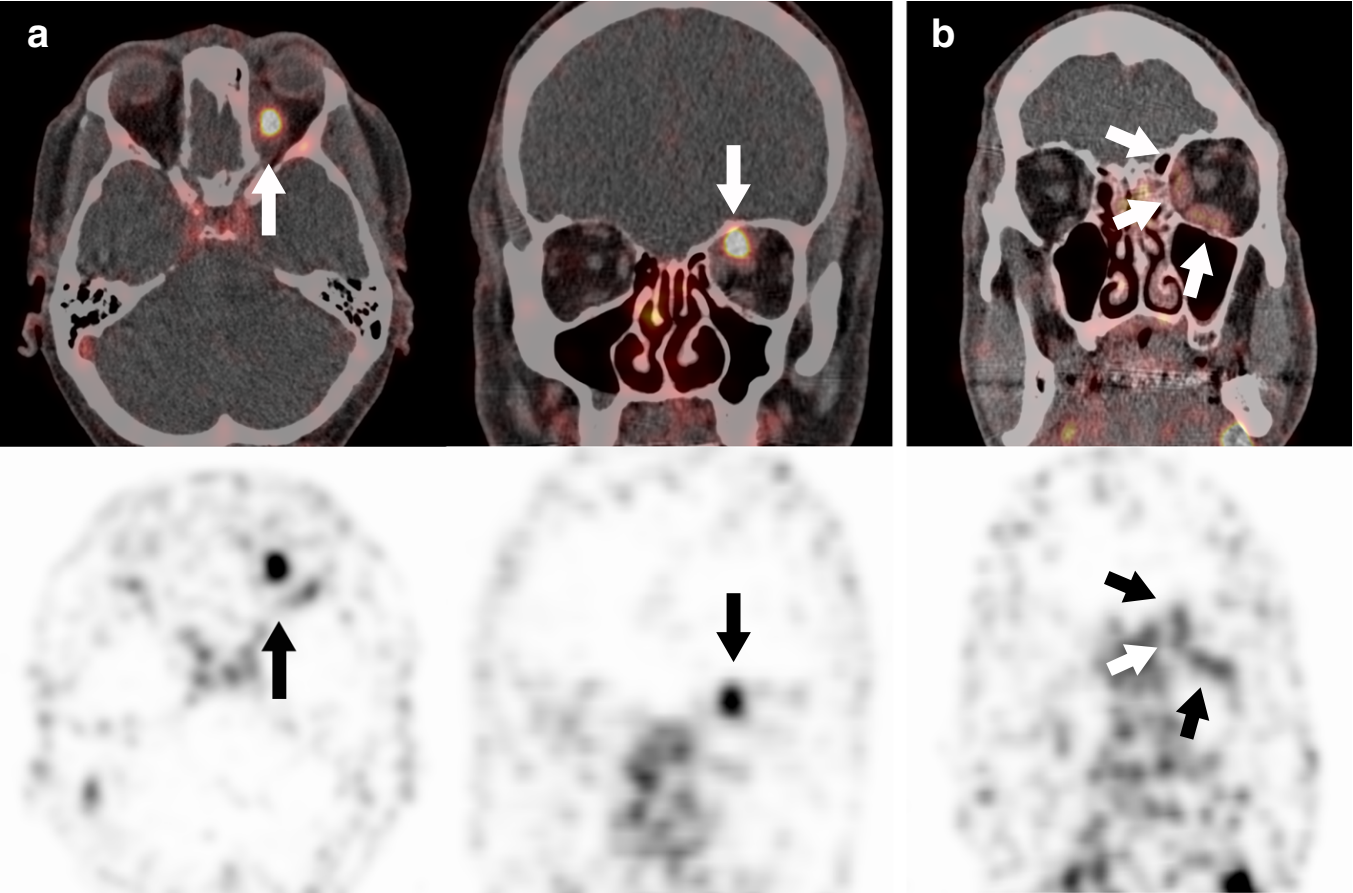
Examples of 89Zr-rituximab PET/CT uptake. **a** Strong uptake in fusion image and PET-only image for the axial and coronal planes (arrows point at the lesion, SUVmax> 2.0; case 6). **b** Moderate uptake in fusion image and PET-only image (arrows point at affected muscles, SUVmax 1.0–2.0, case 5)

**Fig. 2:**
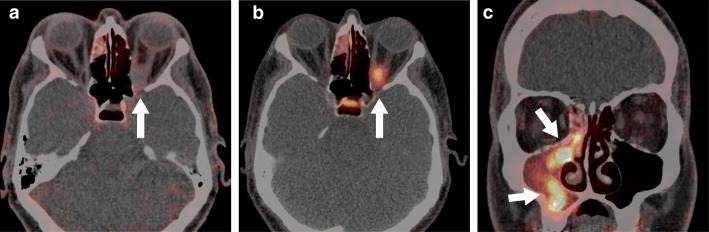
Patient with a meningioma in the left orbital apex and sinusitis of the right maxillary sinus (case 8). **a** Negative ^89^Zr-rituximab PET/CT (white arrow, SUVmax < 1.0). **b** Positive 68Ga DOTA-TATE PET/CT, white arrow. **c** ^89^Zr-rituximab uptake in a co-existing sinusitis at the contralateral side, white arrows

In six patients, we found a high ^89^Zr-rituximab PET uptake (standardized uptake value (SUV) > 2.0) within the orbital masses (Table 1, example in Fig. 1a). One patient with a strong PET uptake had an active TED with CAS 5. In two patients, a moderate ^89^Zr-rituximab uptake was seen, of which one patient had an IOI in the orbital apex and one patient was diagnosed with IgG4+ ROD (Fig. 1b). In four patients, there was no pathological ^89^Zr-rituximab uptake. We diagnosed patients with a negative scan as myositis (two patients), active TED with CAS 4, and meningioma in the orbital apex (case 8) which became most likely after a positive Gallium-68-labelled DOTA-TATE PET/CT (Fig. 2b), as meningiomas usually have a high somatostatin receptor expression [12]. In this last patient, a concurrent sinusitis of the right maxillary sinus coincidentally showed a strong ^89^Zr-rituximab uptake (Fig. 2c). Interestingly, IOI located in the lacrimal gland or diffuse within the orbit had strong uptake of ^89^Zr-rituximab, whilst myositis and masses of the apex had moderate to no uptake. Strong ^89^Zr-rituximab uptake was inhomogeneous with a focal intense uptake within the lesion.

### Histopathology

Eight patients had an open incisional biopsy through a lid crease or swinging-eyelid surgical approach, dependent on the localization within the orbit [3]. For all but one (case 5), the histological images were not suggestive for IgG4-related disease. Histopathological examination in seven biopsies showed signs of a chronic inflammation with a lymphoplasmacytic infiltrate, characterized by both CD20+ B cells and CD3+ T cells. CD138+ plasma cells had a polytypic lambda and kappa distribution and either a predominant IgG or IgA differentiation for orbital and lacrimal gland tissue, respectively. In one biopsy, we only found fatty tissue without inflammation due to sampling error and a deep orbital localization.

### Rituximab treatment

**Fig. 3:**
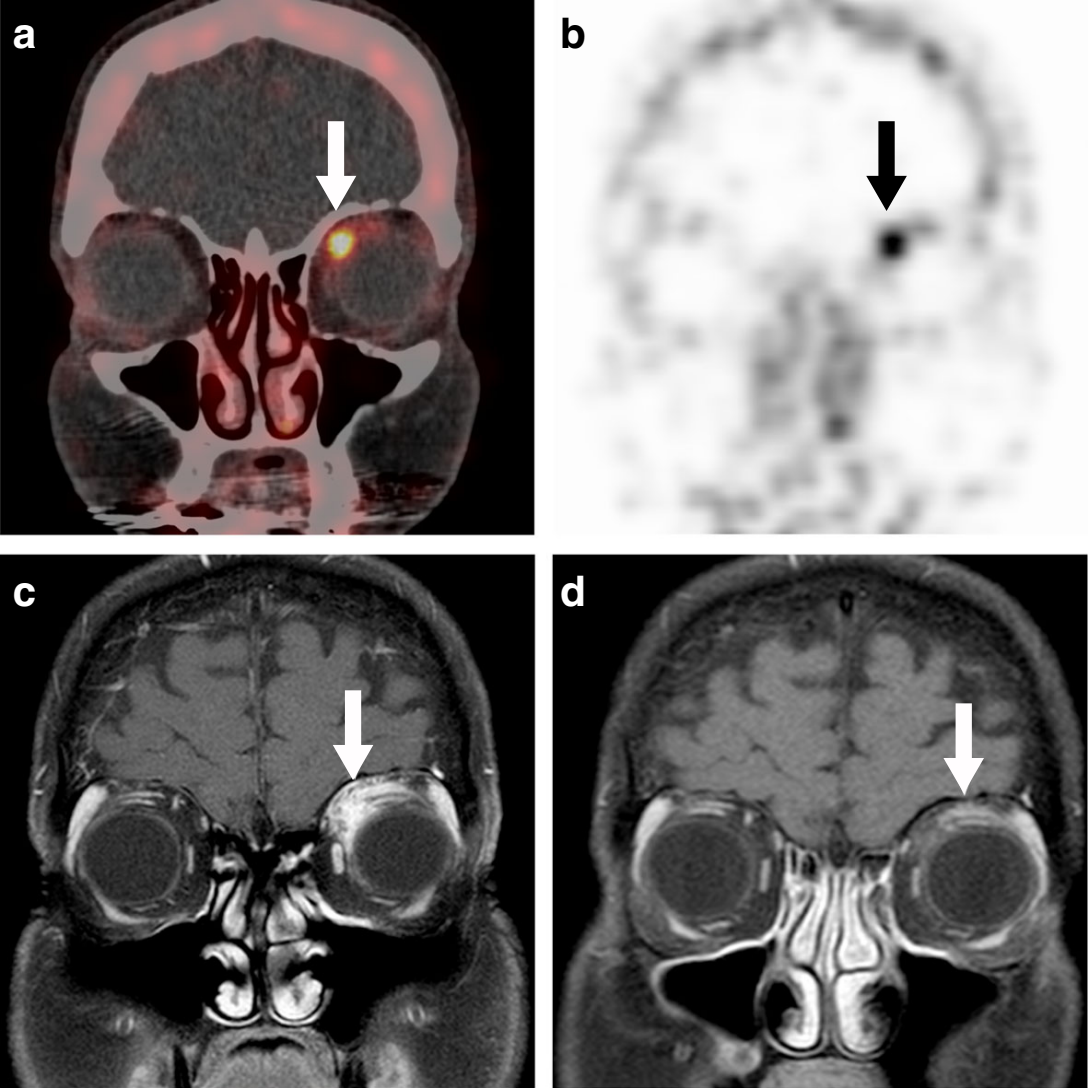
Rituximab treatment response (case 7). **a** ^89^Zr-rituximab PET/CT fusion image with focal uptake in the lesion, white arrow points at the lesion. **b** PET only image, black arrow points at the lesion. Initial (**c**) and post-treatment MRI at 3 months after treatment (**d**), illustrating treatment response

Four patients were treated with rituximab (2 × 1000 mg IV, MabThera, Roche) after a positive ^89^Zr-rituximab PET/CT. All treated patients showed improvement of complaints within the first weeks after treatment. This effect was temporary in 3 out of 4 cases as some of the complaints returned to lesser extent. In one case, all complaints were initially gone (vision > 20/20, no pain and no diplopia), but some complaints returned 6 months later (case 7). A second rituximab treatment was given after 6 months and the patient responded well with remission of complaints. Three other cases (case 9–11) had a significant reduction of complaints (improvement of visual acuity to 20/20, mild or no pain and improvement of eye motility). Of these, one received a second rituximab dose 4 months later (case 10), one had a relapse after 2 months and received additional treatment of oral prednisone (15 mg) and azathioprine (150 mg) (case 11), and one is currently monitored over 6 months whilst still improving (case 9). Two patients had an MRI scan after treatment with rituximab and both showed major radiological improvement with almost complete regression of the mass (case 7 and 9 Fig. 3a–d).

### Other treatments

Two patients with a positive scan did not receive rituximab treatment. One patient improved after treatment with oral prednisone and receiving infliximab for coexisting Crohn’s disease (case 3) and the other patient improved on IV steroids (case 6). The latter patient was previously treated with oral prednisone for more than 12 months and had a biopsy after ^89^Zr-rituximab PET/CT was performed. With a confirmed diagnosis of IOI, this patient was therefore first treated with IV steroids. Patients with a negative scan were not treated with rituximab and had stable disease, refractory to treatment. The patient with IgG4+ ROD (case 5) had a good response after additional IV steroids were given.

## Discussion

We describe our experience of ^89^Zr-rituximab PET/CT in 12 patients suspected of refractory orbital inflammation within the University Medical Center Utrecht. We have found a strong ^89^Zr-rituximab uptake in orbital inflammatory diseases of the lacrimal gland and as a mass or diffuse within the orbit. Idiopathic myositis and involvement of the orbital apex showed ^89^Zr-rituximab uptake to a lesser extent. A focal density was found in masses with a strong uptake. All four patients treated with rituximab after a positive ^89^Zr-rituximab PET/CT had a good response during one or multiple treatments.

Five studies previously investigated the use of ^89^Zr-rituximab PET/CT in humans with different disease entities. Two studies investigated B cell lymphoma in a total of 11 patients [7, 13]. Lymphoma masses showed ^89^Zr-rituximab PET uptake that was greater in the tumour mass without a preload of unlabelled rituximab [7]. The tumour uptake of labelled rituximab correlated with the in-tissue CD20 expression [13]. One study investigated the predictive value of ^89^Zr-rituximab PET/CT for the effectiveness of rituximab treatment in rheumatoid arthritis patients [5]. A case report described the use of ^89^Zr-rituximab PET/CT in the diagnostic process of neurolymphomatosis in the sciatic nerve [6]. Finally, one study investigated three patients with multiple sclerosis, reporting no penetration of ^89^Zr-rituximab in the brain [14].

Besides Zirconium-89, intact CD20 labelling with rituximab has been performed with Iodine-124 [15] for patients with rheumatoid arthritis and Technetium-99m [16] in several inflammatory conditions, showing feasibility for CD20 imaging. However, Zirconium-89 remains the most suitable for internalizing intact monoclonal antibodies [17]. ^89^Zr-rituximab has a relatively long half-life and high effective dose of approximately 0.5 mSv/MBq [18]. The radiation dose should therefore be considered and balanced to the clinical benefits.

Surgical (open) biopsies are recommended for the diagnosis of orbital masses [3]. Unfortunately, biopsies deep in the orbit can be difficult, not-representative and potentially lead to severe complications to the optic nerve and extra-ocular muscles [4, 19]. The ^89^Zr-rituximab PET/CT can be of aid in distinguishing inflammatory and lymphoproliferative disorders from other orbital diseases, as we demonstrate in case 8 (Fig. 2). This technique can, therefore, in combination with clinical and laboratory findings [9] and MRI imaging [20], have additional value for a comprehensive diagnosis in difficult cases.

Our study shows that stronger focal ^89^Zr-rituximab intensity can occur within the orbital mass (Fig. 3). It was previously suggested that a higher tumour CD20+ expression correlated with a higher PET/CT intensity in lymphoma patients [13]. In our opinion, representable biopsies should yield the densest area of inflammatory cells within the tumour to provide the most information and exclude lymphoma. The yield of the inflammatory area within the biopsies was dependent on the morphology of the inflammation within the normal tissue as well as the depth of the mass within the orbit, reflecting the difficulty of an orbital biopsy. Because we show focal uptake in this study, we believe that the ^89^Zr-rituximab PET/CT could be used as pre-biopsy orientation for targeting higher intensity areas during incisional biopsies for difficult cases.

The role of rituximab as a treatment in refractive orbital inflammatory disease is currently under investigation. Previous case reports and a phase I/II trial have indicated a strong potential for the effectiveness of rituximab treatment in refractory orbital inflammation [8, 21]. However, not all patients benefit from this treatment, and the non-responders have an unnecessary exposure to potentially severe adverse effects. For IOI, the presence of a mixed B cell and T cell profile in the histopathological analysis of the masses reflects the involvement of both cells in the pathogenesis of the disease [22]. Although theoretically logical, there is no evidence of better rituximab effectivity in IOI patients with a more profound B cell involvement. In TED, varying results have been published of the effectiveness of rituximab treatment [23–27]. Most early reports and a randomized controlled trial [24] describe clinical improvement with treatment in almost all patient, while another randomized controlled trial did not show an overall improvement compared to placebo. The search for factors that can predict the effectiveness of rituximab in TED and other orbital inflammatory diseases continues [28].

The use of ^89^Zr-rituximab PET/CT to predict rituximab treatment response has been demonstrated in a small cohort of rheumatoid arthritis patients by quantification of the uptake [5]. Although limited by the number of patients, we can now extrapolate this theory to orbital inflammatory diseases as we see a good response of rituximab in high-uptake patients in the current study. We would encourage future research investigating the predictive potential of rituximab therapy in inflammatory diseases, including TED, to use ^89^Zr-rituximab PET/CT as an objective and measurable tool.

Several limitations of this study exist, inherent to the retrospective nature. We were not able to include patients diagnosed with a biopsy-proven orbital lymphoma. We would expect a high uptake in lymphoma patients [7, 10] and a comparison with inflammatory orbital conditions is warranted for the potential for differentiation. Also, not all patients were treated with rituximab, including the patients with a negative scan. We could therefore not compare patients with a positive and negative scan for treatment effectivity.

## Conclusion

This study describes our institutional experience with the ^89^Zr-rituximab PET/CT in orbital inflammatory diseases. This technique has the potential to be a powerful tool for the detection of B cell-mediated disease within the orbit and ocular adnexa. By visualizing the CD20+ B cells, it can be a valuable addition to the diagnostic armamentarium for orbital inflammatory disease. Higher focal intensities within an orbital mass were found that can potentially pinpoint the location for more representative surgical biopsies. Patients with a strong ^89^Zr-rituximab PET/CT uptake responded well to rituximab treatment. We encourage further research of this technique in the diagnostic process and to predict rituximab treatment response for orbital inflammatory diseases.

## Data Availability

The datasets generated during and/or analysed during the current study are available from the corresponding author on reasonable request.

## Abbreviations

^89^Zr-rituximab PET/CT: Zirconium-89-labelled rituximab positron emission tomography/computed tomography
CAS: Clinical activity score
CD: Cluster of differentiation
IgG4 + ROD: IgG4+-related orbital disease
IOI: Idiopathic orbital inflammation
IV: Intravenous
TED: Thyroid-associated eye disease
